# Pathological Anatomy

**Published:** 1846-03

**Authors:** 


*Pathological Anatomy.—Extraordinary Fœtus.*—A male child was born with all the small intestines spread out on the abdomen. The delicate membrane, a prolongation of the peritoneum, which originally enclosed them as in a sac, had been ruptured in the delivery. The umbilical orifice, through which the intestines passed, with the navel string, was as large as a silver-mark (en sölv-mark,) and portions of them were so strongly adherent to the margin of the orifice, as to render their reduction impracticable. The child died twenty-eight hours after birth. On examination, no room for the intestines was found in the abdominal cavity, and all the parts were larger than ordinarily seen in a new-born child. That portion of the umbilical cord which traversed the sac, was very slender, but farther towards the placenta it was an inch in thickness.—*Lond. Med. Times.*

